# *De novo *7p partial trisomy characterized by subtelomeric FISH and whole-genome array in a girl with mental retardation

**DOI:** 10.1186/1755-8166-4-21

**Published:** 2011-10-03

**Authors:** Aswini S, Venkata O Padmalatha, Saranya G, Durgadatta T, Raseswari T, Kanakavalli M Kulashekaran, Meena J, Chandra N, Lalji S, Lakshmi R Kandukuri

**Affiliations:** 1 Department of Genetics, Dr. ALMPG. Institute of Basic Medical Sciences, University of Madras, Taramani, Chennai - 600113, Tamil Nadu, India; 2 Clinical Research Facility-Medical Biotechnology, Centre for Cellular and Molecular Biology Annex II, Uppal Road, Hyderabad - 500007, Andhra Pradesh, India; 3 Genome Foundation, Centre for Cellular and Molecular Biology, Hyderabad-500007, India; 4 Department of Medical Genetics, Institute of Obstetrics and Gynecology, Madras Medical College, Government Hospital for Women and Children, Egmore, Chennai - 600008, India

## Abstract

Chromosome rearrangements involving telomeres have been established as one of the major causes of idiopathic mental retardation/developmental delay. This case of 7p partial trisomy syndrome in a 3-year-old female child presenting with developmental delay emphasizes the clinical relevance of cytogenetic diagnosis in the better management of genetic disorders. Application of subtelomeric FISH technique revealed the presence of interstitial telomeres and led to the ascertainment of partial trisomy for the distal 7p segment localized on the telomeric end of the short arm of chromosome 19. Whole-genome cytogenetic microarray-based analysis showed a mosaic 3.5 Mb gain at Xq21.1 besides the approximately 24.5 Mb gain corresponding to 7p15.3- > pter. The possible mechanisms of origin of the chromosomal rearrangement and the clinical relevance of trisomy for the genes lying in the critical regions are discussed.

## Introduction

Unbalanced rearrangements leading to chromosomal and thereby genomic imbalance have been traditionally associated with abnormal phenotypes. While rearrangements involving sex chromosomes may not be linked to severe motor impairment, those involving autosomes may result in dysmorphic facies, malformations and mental retardation (MR). Further, the detection of chromosome ends in majority of the translocations has emphasized the role of telomeric rearrangements as the cause of several genetic diseases. The telomeric regions of human chromosomes are enriched for CpG islands and have been reported to have the highest gene density in the entire genome [1].

Telomeres composed of the hexameric TG-rich repeat (TTAGGG)n sequences and ranging from 2 to 15 kb in length, cap the ends of mammalian chromosomes. They are very essential for the stability and integrity of the chromosomes by preventing end to end fusion and exonucleolytic degradation. Additionally, they also play a role in cell longevity by actively participating in the complete replication of DNA [2]. Telomeres are synthesized by telomerase, a reverse transcriptase-like enzyme that is composed of RNA and protein catalytic subunits [3]. The telomeric sequences serve as binding sites for a specialized set of proteins that give rise to a unique chromatin structure [4] and assist in chromosome attachment to the nuclear matrix [5] and in segregation of chromosomes at meiosis [6].

Interstitial telomeric sequences could result from i) termino-terminal rearrangements with fusion of the telomeres of two chromosomes, ii) rearrangements in which an acentric fragment of one chromosome fuses to the telomere of another chromosome and iii) telomere-centromere rearrangements in which telomeric sequences of one chromosome fuse with the centromere of another chromosome [7]. There is increasing evidence that interstitial telomeres may be hot-spots for chromosomal instability leading to an increased rate of spontaneous and induced chromosomal rearrangements such as translocations, ring chromosomes, and unstable jumping translocations seen in tumor cells [8,9]. These sequences are also present in certain intrachromosomal sites e.g., within the band 2q13 and have been proposed to have originated as a result of telomeric fusions in the course of genome evolution [10]. In some cases, interstitial telomeric sequences can be considered as non-functional elements analogous to the inactivated centromeres in dicentric chromosomes [11,7]. Another case of 7p partial trisomy syndrome associated with interstitial telomeres is added to the existing literature through this report stressing the fact that rearrangements involving telomeres or telomere dysfunction can handicap a cell severely and might lead to genetic disease.

## Case report

The proband was a 3-year-old female child born to non-consanguineous parents. Her mother was 29 years old and her father was aged 32 years at her birth. She weighed 2.25 kg at birth and was delivered normally after an uneventful pregnancy. The child was referred for cytogenetic analysis with a complaint of severe developmental delay and dysmorphic facies. Clinical examination revealed open anterior fontanelle, hypertelorism, low set ears, squint eyes and clinodactyly of both little fingers. CT scan indicated mild cerebral atrophy. She was afflicted with bronchopneumonia and did not exhibit any organomegaly. T_4 _and T_3 _levels were within normal limits. Pedigree analysis did not indicate any genetic disorder or congenital defects in any of the family members.

## Materials and methods

### Cytogenetic analysis

Chromosomal preparations were obtained from PHA-stimulated lymphocytes of the proband, her parents and her elder brother following the protocol of Hungerford (1965) [12] and were GTG-banded [13]. High resolution banding was performed by using ethidium bromide [14]. This study was approved by the Institutional Ethics Committee. Twenty-five metaphases were analyzed from each individual. About five well-banded metaphases were documented and karyotyped using Applied Spectral Imaging Systems karyotyping software (BandView version 5.5). Chromosomal anomalies were designated using standard nomenclature (ISCN 2009).

### M-FISH analysis

M-FISH was performed on unbanded chromosome preparations from proband using 24Xcyte Human M-FISH probe kit from MetaSystems, Gmbh, Germany, following the manufacturer's specifications. Images were captured under respective filters using Zeiss Axioscope microscope (Zeiss, Jena, Germany). Image processing and analysis were done with the ISIS imaging system (MetaSystems, Gmbh, Altlussheim, Germany).

### FISH analysis using WCP probes

Targeted FISH procedures were performed using whole chromosome painting probes for chromosome 7 (WCP7, FITC, MetaSystems, Gmbh, Germany) and chromosome 19 (WCP 19, Spectrum Green, Vysis Inc., USA) according to the manufacturer's instructions.

### FISH analysis using subtelomere probes

FISH experiments were performed using ToTelVysion™ subtelomere multicolour DNA probe Mixture 7 (TelVysion 7p Spectrum Green, 7q Spectrum Orange, TelVysion 14q Spectrum Orange and Spectrum Green) and Mixture 14 (TelVysion 19p Spectrum Green, 19q Spectrum Orange, LSI 19p13 Spectrum Aqua) (Vysis Inc., USA) to confirm cryptic subtelomeric rearrangements. About twenty-five metaphases were captured under Zeiss Axioscope microscope and analysed using ISIS imaging system (MetaSystems).

### Whole-genome cytogenetic microarray-based hybridization analysis

DNA was extracted using the Nucleon BACC2 DNA extraction kit (Amersham Pharmacia Biotech, Piscataway, NJ) following the manufacturer's protocol. Microarray analysis was performed using the Affymetrix^® ^Whole-Genome Cytogenetic 2.7 M Array which detects both known and novel chromosome aberrations across the entire genome. Detailed analysis was done using Affymetrix^® ^Chromosome Analysis Suite (ChAS) software ver. 1.1. (Affymetrix INC. Santa Clara, CA, USA) and UCSC Genome Browser Assembly was used for localization of gains and losses in the copy number analysis.

## Results

### Cytogenetic analysis

**Figure 1:**
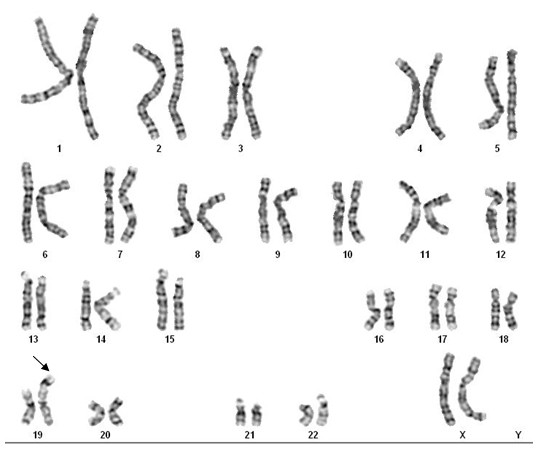
**GTG-banded karyogram of the proposita showing an abnormal chromosome 19**.

Chromosomal analysis revealed an abnormal chromosome 19 with no evidence of mosaicism in the proband who was found to have the karyotype 46, XX, der(19)t(19;?)(p13;?) (Figure 1). The origin of the terminal dark band could not be determined as the chromosomal constitution of her parents was normal. Her brother also exhibited a normal karyotype.

### FISH analysis

**Figure 2:**
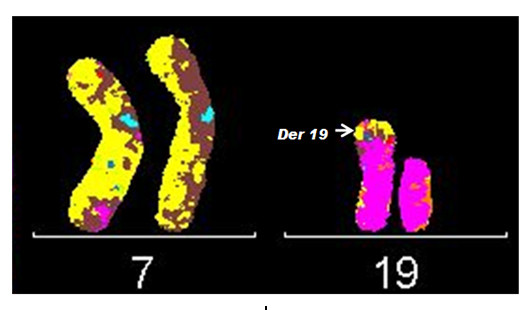
**mFISH reveals the derivative chromosome 19 to contain material from chromosome 7**.

**Figure 3:**
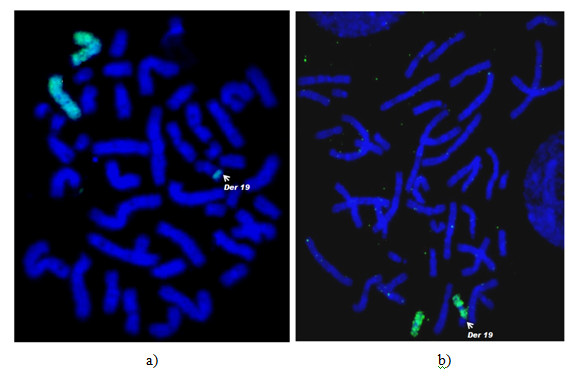
**FISH using whole chromosome paints - a) WCP7 (green) and b) WCP19 (green) probes confirms that the translocated segment on derivative chromosome 19 (indicated by arrow) was derived from chromosome 7**.

**Figure 4:**
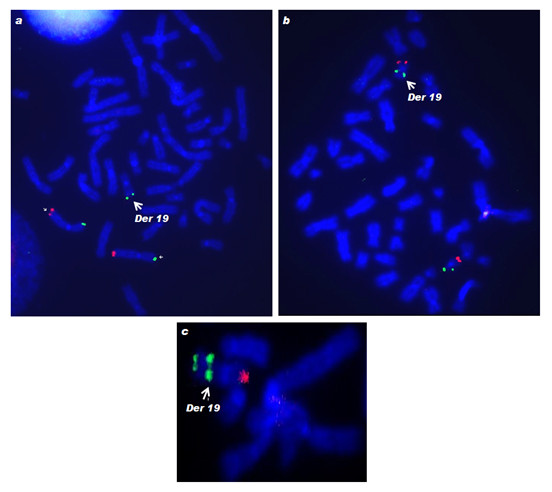
**FISH using subtelomeric probes showing signals on - a) 7p (green), seen also on derivative chromosome 19 (arrow) and 7q (red); b) 19p (green) and 19q (red); and c) interstitial 19p signal near the breakpoint junction (green, indicated with an arrow), 19q (red) and 7p (green) on the der(19)t(7;19) chromosome**.

M-FISH allowed the precise identification of the extra material on the derivative chromosome 19 to be a segment of chromosome 7 (Figure 2) and this was confirmed employing WCP7 and WCP19 probes (Figure 3a and 3b). Although M-FISH and FISH with WCPs did not reveal the arm of chromosome 7 involved in the rearrangement, FISH using subtelomere probes showed the presence of three sets of subtelomeric sequences within the abnormal chromosome i.e., subtel 19p, 19q and 7p (Figure 4a-c), substantiating the partial trisomy of chromosome 7p. The karyotype was consequently determined to be 46, XX, der(19)t(7;19)(p15;p13.3).

### Whole-genome microarray cytogenetic hybridization analysis

**Figure 5:**
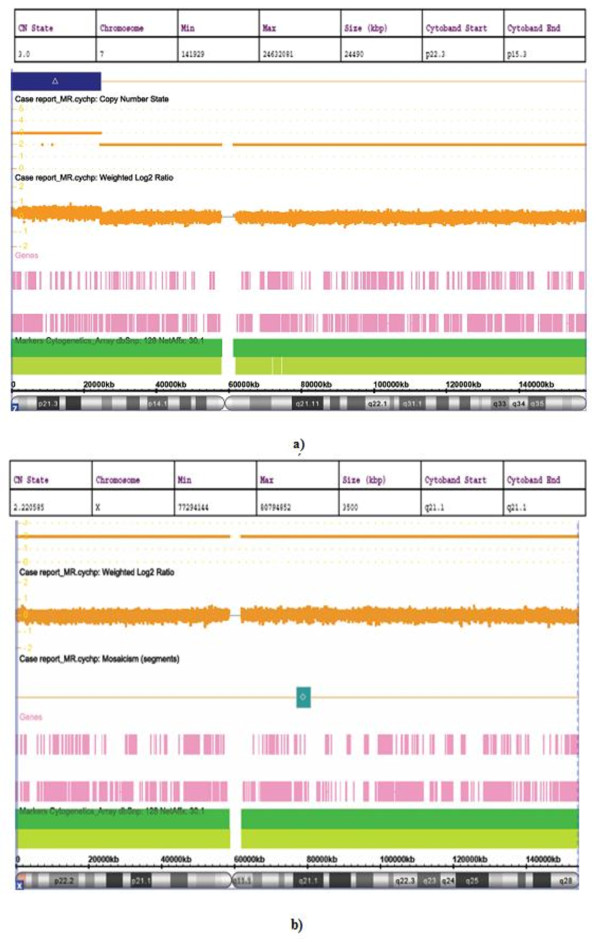
**Whole-genome microarray analysis with respective segment report showing a) copy number gain "partial trisomy" of 24490 kb corresponding to cytobands 7p15.3- > pter b) mosaic gain of 3500 kb on Xq21.1**.

Array-based comparative genomic hybridization was used for high-resolution mapping of the breakpoints that were identified using conventional and fluorescence techniques. A copy number gain ('partial trisomy') of 24490 kb was observed for the short arm of chromosome 7 involving the bands 7p15.3-pter (chr7: 141029-24632081, hg18) (Figure 5a). Further, an apparent mosaicism for a gain of 3500 kb corresponding to Xq21.1 (chrX: 77294144-80794852, hg18) was also noted (Figure 5b).

## Discussion

Loss of telomere or telomere function can lead to breakage-fusion-bridge events that involve repeated fusion and breakage of chromosomes thereby leading to instability, rearrangements including unbalanced translocations and terminal deletions, gene amplification and subsequently cancer [15,16]. Generally chromosomes harbouring a break at their ends stabilize themselves through telomere capture which is telomerase-independent by acquisition of a pre-existing telomere through recombination or via telomere healing which is telomerase-dependent by *de novo *synthesis of a new telomere [17,18]. Another possibility is that the telomere cap structure might be disrupted without chromosome breakage, through the unfolding of a putative hairpin loop or the transient deficiency of telomere-binding proteins. This would permit the chromosome end to be joined to a broken segment of another chromosome and thus would give rise to a non-reciprocal exchange [11,9].

Lack of whole chromosome paint probe WCP19 signal on chromosome 7 confirmed the absence of a balanced translocation in the proposita. Therefore, chromosome healing through telomere capture appears to be the most probable mechanism of genesis of the derivative chromosome 19 where the acentric fragment of chromosome 7 fuses to the telomere of chromosome 19. The presence of subtelomeric 19p probe signal on the derivative chromosome indicates that the break had not occurred in the subtelomere but in the distal part removing all or some of the telomeric consensus sequence although this could not be validated. Telomere capture always occur *de novo *in contrast to unbalanced cryptic translocations which are mostly inherited from balanced carrier parents [9]. The rearrangement observed in the proband was also *de novo *in this study. The derivative chromosome could have been the outcome of a *de novo *event arising during gametogenesis from either of the parent. Baird *et al. *(2006) [19] have elucidated the association between telomere truncation events and the male germ cell line. Such events may be influenced by paternal age at conception [20] and have the potential to generate chromosomal rearrangements and to limit replicative potential [19]. However, this was not a case of advanced paternal age.

The deleted chromosome in the proposita could have been repaired by acquiring the end of chromosome 7p, while the distal end of the broken chromosome 19p and the now broken donor chromosome 7 remains unrepaired. Thus an unbalanced segregation of a parental nonreciprocal translocation at meiosis is the most common mechanism leading to partial trisomy. This process involves recombination-dependent DNA replication termed break-induced replication (BIR) [21]. This mechanism has been observed in yeast and is widely hypothesized to be the etiology behind telomere associations in mammalian cells too. Although such healing events potentially give rise to chromosomes carrying terminal deletions and a new telomere, it allows the truncated chromosome to replicate and segregate normally [22].

The abnormal phenotype observed in the proposita might be partly attributed to telomeric position effect (TPE), a silencing mechanism involving telomere architecture and classical heterochromatin features, and partly due to trisomy 7p15-pter. Repositioning of active genes near telomeres or subtelomeric sequences as a result of chromosome rearrangements may pave way to TPE consequently playing a direct role in human genetic diseases including mental retardation. The subtelomeres are usually prone to extensive recombination and may either buffer or facilitate the spreading of silencing that originates from the telomere [23,24].

A survey of cases of 7p partial trisomy reported in the literature reveals a well-defined pattern of musculoskeletal, cardiovascular, neurological, genital, and ocular abnormalities in decreasing order of frequency. Further, approximately one-third of the patients were described to have died in infancy and one-half had severe mental retardation [25]. The first case was reported by Willner *et al. *(1977) [26] who also stressed the potential of the derivative chromosome in regional gene mapping. The most striking effect of this anomaly is found to be a severe failure in cerebral development resulting in gross retardation. The clinical findings observed in the proposita including psychomotor retardation, large anterior fontanelle, hypertelorism and low-set ears were in accordance with these earlier reports (Table 1). However, congenital heart defects and skeletal abnormalities [27,28] were not recorded in the index case. This difference may be explained by the exact position of the breakpoints at the molecular level, individual gene dosage and also probably TPE.

**Table 1:** Clinical features seen in individuals with 7p partial trisomy reported in the literature

Karyotype	Clinical features
46, XY, der(22)t(7;22)(p21;q13)pat	Retarded mental and motor development, congenital heart abnormalities, high broad palate, spilt uvula, microbrachycephaly, increased transillumination of the frontal area of the skull, right ventricular hypertrophy, atrophy of the brain [27].

46, XX, der(5)t(5;7)(p15;p15)pat	A 4-month-old female infant with asymmetric cranium with widely patent anterior fontanelle and metopic suture, deep widow's peak, hypertelorism, bilateral choanal atresia, prominent nasal bone, low set and rotated ears, maxillary hypoplasia, high arched palate, micrognathia, arachnodactylty with contractures of the interphalangeal joints, congenitally dislocated hips, vertical talus [26].

46, XX, ?der(22)t(7;22)(p15;q13)pat	Hypotonic, elongated skull with a widely separated metopic suture, microphthalmos, mongoloid slant, skeletal abnormalities such as arachnodactyly, flexion deformity of the wrists, talipes calcaneo-valgus, and widely separated first and second toes; unilateral, single palmar crease; died at 8 weeks. Necropsy study showed hydrocephalus and microgyria, hypertrophic left ventricle with persistent ductus arteriosus, bilateral cystic kidneys [28].

46, XX, der(22)t(7;22)(p15;q13)pat [Sib of the previous case]	A long face with narrow palpebral fissures, with a slight mongoloid slant to the eyes, epicanthic folds, broad and flat nasal bridge, high and arched palate, prominent maxilla with a thick lower lip, slight weakness of the left arm and shoulder, remained extremely retarded at the age of 9 years [28].

46, XX, der(21)t(7;21)(p15;p12)mat [41]	Not available

46, XY, der(11)t(7;11)(p15;q25)mat 46, XX, der(11)t(7;11)(p15;q25)mat	Psychomotor retardation, growth retardation after birth, wide anterior fontanel, left esotropia, sacral dimple, bilateral undescended testis, whorls on six fingers, unilateral palmar transverse crease, bilateral high axial triradius Fetus conceived subsequently with micrognathia; aborted [42].

Three girls with partial trisomy 7p from two families with balanced translocations involving 7p	Several congenital malformations - heart defects, cleft palate, postaxial polydactyly, choanal stenosis/atresia [43].

46, X, der(X)t(X;7)(q28;p15)	Hypotonic and severely retarded child, asymmetric face, slight hypertelorism, antimongoloid slant, strabismus, exophoria, exotrophia, low set ears, high arched palate, joint laxity, bilateral allux valgus, bilateral flat feet, genu valgum recurvatum, alive at 11 years [44].

46, XY, der(8)t(7;8)(p15;p22)	A 6-year-old boy with high and large forehead flattened at the centre due to the abnormally large and persistent gaping anterior fontanelle and (sagittal) metopic sutures, consequent hypertelorism, broad nasal bridge, cutis laxa, often denounced by folded neck, joint, cardiovascular anomalies, psychomotor delay, clubfoot, a possible typical dermatoglyphic pattern [45].

46, XY, der(9)t(7;9)(p21.2;p23.5) (Back *et al*.1997)	Not available

46, XY, der(21)t(7;21)(p21.2;q22.3)mat	1-year-old boy with mental and physical retardation, a large anterior fontanel, brachycephaly with flat occiput, short and stubby fingers, generalized hypotonia, ocular hypertelorism, low nasal bridge, long philtrum, high-narrow palate, apparently low-set ears, and a small mandible [46].

46, XX, der(9), t(7;9)(p15;p24)	Generalized developmental deficits, high and large forehead, hypertelorism, broad nasal bridge, hypothyroidism, obesity, cerebral palsy [25].

46, X, der(Y)t(Y;7)(p11.32;p15.3)	A three-month-old boy with growth deficiency, postnatal microcephaly with large fontanels, wide sagittal and metopic sutures, hypertelorism, choanal stenosis, micrognathia, bilateral cryptorchidism, hypospadias, abnormal fingers and toes, severe developmental delay [32].

Normal development is strongly associated with gene dosage and it is established that an imbalance leads to abnormal phenotype. The majority of genes exhibiting dosage sensitivity are regulatory which affect the expression of vital target genes [29,30]. Rapid progress in mapping of candidate genes has led to the identification of several loci involved in neurological dysfunction and, in particular, mental retardation. Array-based comparative genomic hybridization confirmed partial trisomy 7p (7p15.3-pter) which harbors about 130 protein coding genes and five of these genes found to encode transcription factors could be crucial in the neuronal pathway (Table 2). Two genes *P2PRY10 *and *BRWD3 *of 11 genes on the cytoband Xq21.1, found to show copy number variation in mosaic form, could also have a possible role in the causation of mental retardation. Triple dosage of the *TWIST *gene on 7p21.1-p21.2 has been attributed to abnormal skull development due to delayed closure of fontanelles [31,32].

**Table 2:** Function of genes of significance localized in the regions 7p15.3- > pter and Xq21.1 found to be altered in microarray analysis.

Cytoband	Gene	Function
7p15.3	*NPY*	Encodes a neuropeptide, widely expressed in the brain and autonomic nervous system, which functions through G protein-coupled receptors to inhibit adenylate cyclase, activate mitogen-activated protein kinase, regulate intracellular calcium levels and activate potassium channels, thus have an effect on neuronal excitability and synaptic transmission.

7p21.1	*FERD3L*	Its protein is expressed in the developing CNS and functions as a transcriptional inhibitor, thus a negative regulator of neurogenesis [47].

7p21.1-p21.2	*TWIST1*	Acts as a transcriptional regulator and is involved in membranous ossification occurring during frontal, parietal, and malar bone formation; triple dosage of this gene might be responsible the delayed closure of a large anterior fontanelle.

7p22.1	*RADIL*	Downstream effector of Rap required for cell adhesion and migration of neural crest precursors during development

7p22.1	*DAGLB*	Required for axonal growth during development and for retrograde synaptic signalling at mature synapses [48].

Xq21.1	*P2RY10*	P2Y receptors are G-protein-coupled receptors and their activation initiates a wide range of signaling cascades including PLCbeta, PLD, PLA2, AC and MAPK/MEK kinase. They have diverse physiological roles including regulation of platelet aggregation, muscle contraction, neurotransmission and epithelial cell communication and migration.

Xq21.1	*BRWD3*	Mutations in *BRWD3 *cause mental retardation X-linked type 93, which is also referred to as mental retardation X-linked with macrocephaly.

Neuronal migration disorders, in which newly born neurons fail to migrate correctly from the ventricular zone to their final neocortical positions, are associated with neurological dysfunction [33]. Mental retardation could reflect an abnormal dosage effect of *NPY *gene during brain development. The neuropeptide Y, a neurotransmitter found in the CNS, functions through G protein-coupled receptors (GPCR) and influences many physiological processes including cortical excitability [34,35]. The P2Y purinoceptor 10 protein, encoded by the *P2PRY10 *gene localized on Xq21.1, is itself is a GPCR and is also known to have a role in neurotransmission. Both the genes activate MAPK/MEK signaling pathway.

Another gene mapping in the partial 7p trisomic region is *RADIL *(RA [Ras association] and DIL domains) and its product is known to play a critical role in cell adhesion and migration [36]. Knockdown studies of *radil *in the zebrafish model presented multiple defects in neuronal cell (NC)-derived lineages such as cartilage, pigment cells and enteric neurons [37]. RADIL, a downstream effector of RAP, a member of RAS family also activates MAPK/MEK signaling pathway. Murine models indicate a key-role for RAS signaling in memory, learning and cognitive abilities [35,38]. Human constitutional defects associated with NC function account for up to 30% of all congenital birth defects [39] which often include craniofacial abnormalities.

**Figure 6:**
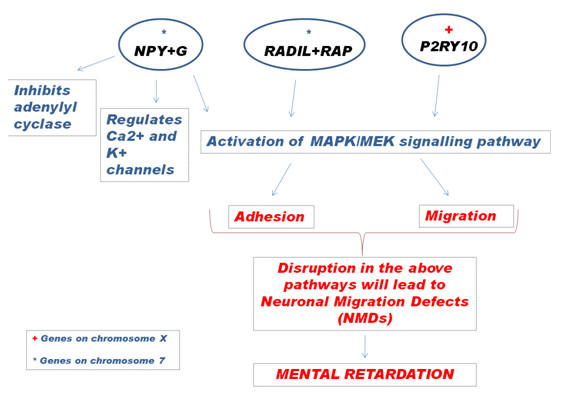
**A hypothetical schematic representation of the interaction of genes *NPY *and *RADIL *on 7p15.3- > pter and *P2RY10 *on Xq21.1**.

Mental retardation frequently occurs in patients with disorders of the RAS/MAPK signaling cascade [40]. MAPK/MEK activation thus seems to be a crucial event in neuronal adhesion and synaptic functions. The association of the three genes *NPY, P2PRY10 and RADIL *with this critical pathway and the probable cross-talk between them (Figure 6) identifies them as the prime candidates for the observed phenotype.

In conclusion, application of molecular cytogenetic techniques has not only resolved the origin of the abnormality but also has revealed the presence of interstitial telomeric sequences on the derivative chromosome. Further, segmental imbalances thus detected prove significant in phenotype-genotype correlation and in the identification of probable candidate genes.

## Consent

Written informed consent was obtained from the patient for publication of this case report and accompanying images. A copy of the written consent is available for review by the Editor-in-Chief of this journal.

## Competing interests

The authors declare that they have no competing interests.

## Authors' contributions

AS performed the cytogenetic analysis; MJ referred and clinically examined the patient; VOP, RT and KMK participated in laboratory work concerning M-FISH and FISH analyses using WCP & subtelomere probes; SG and DT signed out the Whole-genome cytogenetic microarray-based hybridization analysis results; AS, CN and LRK drafted the manuscript; LS has critically reviewed and approved the manuscript. All authors read and approved the manuscript.
